# Becoming who you are: An integrative review of self‐determination theory and personality systems interactions theory

**DOI:** 10.1111/jopy.12380

**Published:** 2018-04-23

**Authors:** Sander L. Koole, Caroline Schlinkert, Tobias Maldei, Nicola Baumann

**Affiliations:** ^1^ Vrije Universiteit Amsterdam; ^2^ Trier University

**Keywords:** human motivation, human self‐regulation, personality theory

## Abstract

One of the enduring missions of personality science is to unravel what it takes to become a fully functioning person. In the present article, the authors address this matter from the perspectives of self‐determination theory (SDT) and personality systems interactions (PSI) theory. SDT (a) is rooted in humanistic psychology; (b) has emphasized a first‐person perspective on motivation and personality; (c) posits that the person, supported by the social environment, naturally moves toward growth through the satisfaction of basic psychological needs for autonomy, competence, and relatedness. PSI theory (a) is rooted in German volition psychology; (b) has emphasized a third‐person perspective on motivation and personality; and (c) posits that a fully functioning person can form and enact difficult intentions and integrate new experiences, and that such competencies are facilitated by affect regulation. The authors review empirical support for SDT and PSI theory, their convergences and divergences, and how the theories bear on recent empirical research on internalization, vitality, and achievement flow. The authors conclude that SDT and PSI theory offer complementary insights into developing a person's full potential.

This process of the good life is not, I am convinced, a life for the faint‐hearted. It involves the stretching and growing of becoming more and more of one's potentialities. (Rogers, 1961, p. 196)

## 1 INTRODUCTION

What does it take to unlock a person's full potential? Within modern personality science, the study of what people could become, once so poignantly articulated by Rogers (1961), has been kept alive and well by proponents of self‐determination theory (SDT; Deci & Ryan, 2000; Sheldon, 2014). SDT is a comprehensive framework for understanding human motivation and personality that emphasizes people's inherent tendencies toward growth and self‐actualization through the satisfaction of basic psychological needs for autonomy, competence, and relatedness. Over the past three decades, SDT has become a leading paradigm, which has developed through a continuous series of theoretical extensions and innovations, and an impressive number of empirical studies that have systematically tested the tenets of the framework (for an overview, see Ryan & Deci, 2017). SDT has been used to identify universals in human nature and individual differences to map out momentary experiences as well as large‐scale social and cultural processes. Moreover, insights from SDT have been applied around the world, in such important life domains as education, work, close relationships, and psychotherapy.

As students of motivation and personality, we have long been admirers of SDT. Our own work in the area, however, has been guided by personality systems interactions (PSI) theory (Kuhl, 2000a; for an overview, see Baumann, Kazén, Quirin, & Koole, 2018). Like SDT, PSI theory is a comprehensive framework for understanding human motivation and personality. The main difference between the theories is one of perspective: SDT has emphasized a first‐person perspective, which highlights subjective experience as a causal determinant of motivation and personality. By contrast, PSI theory has emphasized a third‐person perspective, which highlights objectively observable, partly unconscious competencies that underlie motivation and personality. Because first‐person and third‐person perspectives are both valid ways of understanding motivation and personality, PSI theory and SDT may be regarded as “sibling theories” (Ryan, 2018, p. 37) with considerable integrative potential (Sheldon, 2018).

In the present article, we seek to bring the integrative potential between SDT and PSI theory into sharper focus. Because of the theories' broad scope, our review is necessarily selective and concentrates on how SDT and PSI theory address the question of what it takes to become a fully functioning person. We borrowed the phrase “becoming a fully functioning person” from Rogers's (1961) personality psychology, where it refers to someone who is mature, responsible, and decisive (see also Kuhl, Quirin, & Koole, 2015). As we use the term, it is loosely synonymous with more mundane expressions such as “developing the person's potential” and the more colloquial “becoming who you really are.” The remainder of this article has four parts. In Section 2, we briefly characterize SDT and PSI theory in terms of their historical background, core propositions, methods, and findings. In Section 3, we examine where SDT and PSI theory converge and diverge. In Section 4, we relate the theories to the empirical domains of internalization, vitality, and achievement flow. Finally, in Section 5, we assess what SDT and PSI theory may learn from each other and how insights from both theories may advance the scientific analysis of how people may become fully functioning persons.

## 2 SDT AND PSI THEORY

Modern personality science has witnessed a proliferation of theories and models that seek to explain a handful of relations among certain aspects of personality, typically some trait and a corresponding set of behaviors. This “small theory” approach has the advantage of allowing for the in‐depth study of personality processes. The risk, however, is that researchers end up with no more than a fragmented understanding of personality. The small theory approach therefore needs to be complemented by a “big theory” approach, that is, comprehensive theories that consider how different processes are jointly coordinated within the person.

SDT (Deci & Ryan, 2000; Ryan & Deci, 2017) and PSI theory (Baumann et al., 2018; Kuhl, 2000a) are two contemporary examples of such comprehensive personality theories or “macro‐theories.” A macro‐theory has at least four distinctive features (see also Ryan, 2018). First, a macro‐theory is an organized structure of ideas that has rich connections with the broader philosophies and intellectual traditions within which it is nested. Second, a macro‐theory offers an integrated account of a set of phenomena with well‐specified mutual relations. This distinguishes a macro‐theory from a meta‐analytic aggregation of findings or a postmodern multiplicity of viewpoints. Third, a macro‐theory's explanations are continually refined and tested through systematic empirical observation, often with methods that are uniquely developed for this purpose. Fourth and last, a macro‐theory is supported by a systematic body of evidence that bears on the validity of its propositions.

In what follows, we review SDT and PSI theory side by side, as two macro‐theories of motivation and personality. Prior reviews discussed SDT and PSI theory separately, which meant that they were largely structured along different conceptual dimensions. The latter makes it difficult to compare the two theories. In the present review, we seek to remove this difficulty by discussing SDT and PSI theory in the same narrative format. Specifically, we characterize SDT and PSI theory in terms of the aforementioned features of a macro‐theory. A shorthand overview of our discussion is provided in Table 1. First, we briefly identify each theory's more distal intellectual roots and recount the proximal historical development of SDT and PSI theory. Second, we consider the core propositions by which SDT and PSI theory explain what it takes to become a fully functioning person. Third, we describe the main methods that have been developed for testing key predictions of SDT and PSI theory. Fourth and last, we review the main empirical findings that the theories have so far generated.

**Table 1:** Macro‐theoretical features of SDT and PSI theory

	SDT	PSI theory
Roots	Distal: Humanistic psychology (since 1950s) Proximal: Social‐psychological experiments on intrinsic motivation (Deci, 1971)	Distal: German volition psychology (1900–1930s) Proximal: Action control theory (Kuhl, 1985)
Core propositions	Every human being has three basic needs: autonomy, competence, and relatedness. When people can satisfy these needs, people enter into an autonomous mode of self‐regulation that fosters intrinsic engagement and well‐being. When need satisfaction is thwarted, people enter into an alienated mode of self‐regulation that fosters inner conflict and reduced well‐being. The balance between autonomous and controlled self‐regulation is determined by the supportiveness of the (social) situation and chronic causality orientations.	Fully functioning persons can form and enact intentions (volitional efficiency) and learn from negative experiences (personal growth). Volitional efficiency requires coordination between planning and action, which is facilitated by changes in positive affect. Personal growth requires coordination between autobiographical memory and elementary perception, which is facilitated by changes in negative affect. Affect regulation may occur via social support or self‐regulatory skills (action orientation), developed through sensitive interpersonal interactions.
Methodologies	Surveys, longitudinal studies, experience sampling, behavioral experiments	Surveys, behavioral experiments, objective measures of personality competencies
Key findings	Psychological need satisfaction predicts intrinsic motivation and well‐being across life domains and cultures. Rewards can undermine intrinsic motivation when people experience them as controlling. Autonomy support promotes internalization of initially unattractive activities. Autonomy orientation is associated with higher intrinsic motivation, more internalized motivation, and greater well‐being.	Demand‐related action orientation predicts efficient formation and enactment of difficult intentions. Threat‐related action orientation predicts better access to autobiographical memory and intuitive detection of semantic meaning. Functional advantages of action orientation emerge especially under stressful conditions. Both types of action orientation are associated with efficient affect regulation.

*Note*. SDT = self‐determination theory; PSI = personality systems interactions.

### 2.1 SDT

#### 2.1.1 Historical development

The historical roots of SDT can be traced to humanistic psychology, a psychological movement that emphasizes people's inherent tendency toward self‐actualization (for an overview, see Schneider, Pierson, & Bugenthal, 2014). During the first half of the 20th century, experimental psychology was dominated by behaviorism, a school of thought that assumed human behavior is passively triggered by rewards and punishments from the environment. Humanistic psychology arose partly as a critique of the behaviorist paradigm. The specific impetus to SDT was provided by social‐psychological experiments in the 1970s, which showed that offering external incentives, like money, could lower people's interest and enjoyment for a task (e.g., Deci, 1971). These findings suggested that human motivation is not entirely externally driven, but rather may arise autonomously from within the person. To deepen the understanding of autonomous motivation, Edward Deci and Richard Ryan and their colleagues began to develop SDT from the 1980s onward (Deci & Ryan, 1985).

Within SDT, people's subjective experience of the situation is treated as the proximal motivational force that shapes their behavioral regulation. For instance, monetary rewards are only presumed to undermine intrinsic motivation when people experience them as controlling (Deci, Koestner, & Ryan, 1999). When people perceive a monetary reward as a sign of respect, their intrinsic motivation may even become enhanced. This emphasis on subjective experience means that SDT emphasizes a first‐person perspective on motivation and personality.

The SDT framework consists of six mini‐theories (Ryan & Deci, 2017). The first mini‐theory is cognitive evaluation theory, which describes how the social environment may help or hinder intrinsic motivation, performance, and wellness (see Deci et al., 1999, for a comprehensive review). SDT's second mini‐theory is organismic integration theory (Ryan & Connell, 1989), which outlines how external regulations may become integrated in the self. SDT's third mini‐theory is causality orientations theory, which explains how more autonomous versus more externally controlled behavior can develop into enduring personality dispositions (Deci & Ryan, 1985). SDT's fourth mini‐theory is basic needs theory, which explains how the satisfaction (or thwarting) of basic psychological needs affects well‐being and vitality (Ryan, 1995). SDT's fifth mini‐theory is goal contents theory, which addresses how the contents of people's goals relate to basic need satisfactions and wellness (Kasser & Ryan, 1996).

Finally, SDT's sixth mini‐theory is relationship motivation theory (Deci & Ryan, 2014), which analyzes the interplay of autonomy and relatedness needs in mutually satisfying relationships.

#### 2.1.2 Core propositions

According to SDT, people naturally develop their full potential when circumstances allow them to satisfy their basic psychological needs. Specifically, SDT distinguishes three psychological needs that are inherent in human nature: autonomy, or the desire to feel volitional rather than controlled and to establish inner coherence; competence, or the need to engage optimal challenges and feel effective; and relatedness, or the need to feel valued and connected with others.

As long as people's basic psychological needs are being met, people's natural tendencies toward growth will emerge, leading to enduring intrinsic engagement, vitality, and wellness. In this autonomous mode of self‐regulation, people are prone to internalize external directives such as goals and social norms, to the extent that these directives are compatible with their personal values. Internalization allows people to feel that they act upon external directives out of their own volition (Sheldon, 2014). In the face of external pressures, however, regulations and values may either remain external or be only partly internalized. Specifically, SDT distinguishes between four levels of internalization: (a) external regulation, when people's behavior is directly controlled by external rewards and punishments; (b) introjection, when people adopt external regulations without fully accepting them as their own; (c) identification, when people consciously value the regulation; and (d) integration, when people bring identified regulations in congruence with their personal values and needs.

When internalization has been forestalled, people may feel conflicted or pressured, leading to drops in intrinsic interest and increases in negative affect. These negative motivational consequences may initially be temporary, given that people are resilient. Prolonged periods of being externally regulated, however, can lead to enduring decreases in well‐being. People may eventually switch to a controlled mode of self‐regulation, in which their strivings become disconnected from their true psychological needs. In the latter case, people may become increasingly sidetracked by alternative, self‐protective tendencies, including the proclivity to dissociate psychological experiences, psychological withdrawal, and narcissistic strivings as compensatory motives for unfulfilled needs.

The cumulative result of people's experiences is reflected in their causality orientation, or chronic tendency to self‐regulate in a more or less autonomous manner (Deci & Ryan, 1985). SDT distinguishes three types of causality orientation. First, people vary in autonomy orientation, or the chronic inclination to seek out opportunities for self‐determination and growth. Second, people vary in control orientation, or the chronic inclination to orient their behavior toward external controls such as deadlines or social norms, and to experience their behavior as largely beyond their volitional control. Third and last, people vary in impersonal orientation, or the chronic inclination to focus on their own inadequacies and not behaving intentionally. The more people acquire an autonomy orientation and the less they acquire controlled or impersonal orientations, the more people should be able to develop their full potential.

#### 2.1.3 Methodology

Because SDT prioritizes a first‐person perspective, the primary source of data for SDT researchers has been people's subjective experience. Indeed, SDT researchers have developed a wide range of self‐report measures that tap into SDT‐based constructs, including need satisfaction, autonomous versus alienated self‐regulation, internalization, and vitality. Through the construction of intersubjectively validated scales—a widely used shortcut within psychology—SDT researchers have been able to transform people's subjective judgments into a third‐person format, which renders them subject to standard quantitative analysis.

One of the most widely used SDT scales taps into “perceived locus of causality” (PLOC; Ryan & Connell, 1989), or the extent to which people view their own behavior as caused by internal factors such as their interests, values, and identities as caused by external factors such as other people's demands or other external necessities. Converging research has shown that the PLOC methodology is valid, and it may even provide a window into intuitive knowledge about the healthiness of a motivational tendency (Sheldon, 2014). For instance, people who experience more autonomous motivation as indicated by the PLOC measure display greater consistency between their conscious goals and implicit, nonconscious motives (Sheldon, King, Houser‐Marko, Osbaldiston, & Gunz, 2007; Sheldon, Prentice, Halusic, & Schüler, 2015; Sheldon & Schüler, 2011).

SDT research has traditionally relied on self‐report surveys administered in a single session (e.g., Kasser & Ryan, 1993). In recent years, however, SDT researchers have increasingly conducted longitudinal studies that span longer periods of time (e.g., Jang, Kim, & Reeve, 2012; Sheldon & Houser‐Marko, 2001). Moreover, SDT researchers have been at the forefront of methodological innovations such as ecological momentary assessment (Shiffmann, Stone, & Hufford, 2008), which involves repeated sampling of experiences in real time, as they are unfolding in people's natural environments (Brown & Ryan, 2003; Huta & Ryan, 2010; Milyavskaya, Inzlicht, Hope, & Koestner, 2015).

SDT researchers have further explored a number of more objective experimental methods. The latter include behavioral assessments of intrinsically motivated behavior (Deci, Eghari, Patrick, & Leone, 1994), memory measures such as recognition accuracy (Sheldon, Arndt, & Houser‐Marko, 2003), and implicit methods such as priming (Burton, Lydon, D'Alessandro, & Koestner, 2006; Levesque & Pelletier, 2003; Ratelle, Baldwin, & Vallerand, 2005) and response time assessment (Sheldon et al., 2007).

#### 2.1.4 Key findings

Since the 1980s, SDT has developed into one of the most prominent theories in contemporary psychology, having been examined in literally thousands of studies. Across these studies, SDT's hypothesized relationships between psychological need satisfaction, intrinsic motivation, and well‐being have been consistently supported (for an overview, see Ryan & Deci, 2017). Empirical support for SDT has been obtained in several important life domains, including work, education, and health behavior. For example, among over 1,700 people from Belgium, China, the United States, and Peru, satisfaction of each of the three SDT needs (i.e., autonomy, competence, and relatedness) was found to predict well‐being, whereas frustration of the three needs was found to predict psychological problems (Chen et al., 2015). Other cross‐cultural studies have similarly confirmed SDT's hypothesized empirical relationships across Western and non‐Western cultures.

The flagship experimental paradigm of SDT research remains the study of the effects of rewards on intrinsic motivation. According to SDT, rewards may lower intrinsic motivation when they make people feel like pawns, and thereby undermine the need for autonomy. The undermining effects of rewards have been confirmed in a quantitative meta‐analysis of 128 studies (Deci et al., 1999). The importance of SDT's competence need is supported by experimental evidence for the positive impact of performance feedback on intrinsic motivation (e.g., Vallerand & Reid, 1984). Extensive evidence in developmental psychology has shown that children who are raised in a warm and loving manner by their caregivers tend to become more autonomously motivated for many tasks and activities later in life (e.g., Grolnick & Ryan, 1989; see also Deci & Ryan, 2014). The latter findings support SDT's idea that relatedness plays a key role in fostering intrinsic motivation.

Finally, research has supported SDT's predicted relations between causality orientation and motivational processes. For instance, people high (rather than low) in autonomy orientation have been found to display higher intrinsic motivation in work settings (Gagné & Deci, 2005) and in laboratory tasks (Hagger, Koch, & Chatzisarantis, 2015). Moreover, people high in autonomy orientation have been found to be less susceptible to the undermining effects of extrinsic rewards than people low in autonomy orientation (Hagger & Chatzisarantis, 2011). Taken together, empirical findings have confirmed that self‐regulation is shaped by the interplay between momentary situational forces and more enduring dispositions of the person, as SDT predicts.

### 2.2 PSI theory

#### 2.2.1 Historical and conceptual development

The academic roots of PSI theory go back to Narziss Ach, a German psychologist who pioneered the experimental study of volition during the early 20th century. Ach conceived of volition as an objective competency (albeit with important correlates in subjective experience) that facilitates the enactment of difficult intentions (Ach, 1905, 1910, 1935). Ach's work was mostly forgotten until the late 1970s, when it was rediscovered by motivation psychologist Julius Kuhl (see Koole & Baumann, 2018, for a biography). Kuhl believed that volition is an essential complement to traditional theories of human motivation, which since Lewin (1926) emphasized that motivation is derived from subjective expectancy–value considerations. During the 1980s, Kuhl formulated action control theory (ACT; Kuhl, 1985), which unites Lewinian motivational forces with Ach's volitional mechanisms. Throughout the 1990s, Kuhl extended his approach into a comprehensive analysis of human motivation and personality, culminating in personality systems interactions (PSI) theory (Kuhl, 2000a, 2001, 2009).

PSI theory seeks to identify the objective competencies (also termed *functional mechanisms* or *personality systems*) that allow people to operate as purposive agents who are endowed with personality. PSI theory thus emphasizes a third‐person perspective on motivation and personality. The theory does not deny that the objective operation of personality systems can be meaningfully related to people's subjective experiences and beliefs. However, the theory maintains that any such meaningful relations should be empirically verified rather than a priori assumed.

PSI theory consists of five interconnected models. First, PSI theory posits a hierarchical model of personality structure (Kuhl & Koole, 2008): Low‐level systems perform the elementary functions of moving and perceiving, which are efficient and automatic, but rigid. High‐level systems are more flexible, but more effortful and slower. The highest levels enable volitional action control and acquisition of lived wisdom. Second, PSI theory has a model of personality dynamics: A key principle here is that changes in positive or negative affect forge collaborations between otherwise incompatible personality systems (Kuhl, 2000a). Affect regulation is hence vital for personality functioning. Third, PSI theory proposes a model of how affect‐regulatory skills may develop through sensitive interpersonal exchanges (Koole & Jostmann, 2004; Kuhl, 2000a). Fourth, PSI theory models personality disorders as chronic fixations on personality systems (Kuhl, 2000b, 2001). Fifth and last, PSI theory models implicit motives as “switchboards” (Baumann, Kazén, & Kuhl, 2010) for establishing coalitions between personality systems that help the person to satisfy the psychological needs for achievement, power, affiliation, and freedom (Baumann et al., 2010).

#### 2.2.2 Core propositions

**Figure 1:**
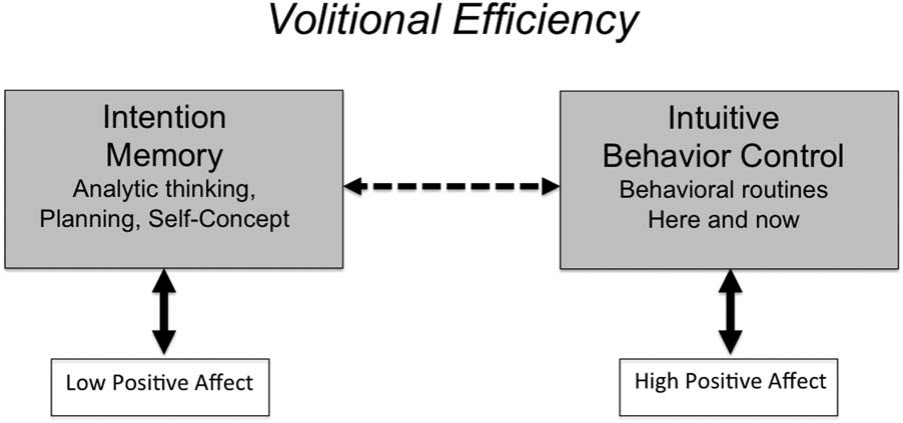
Personality systems interactions for volitional efficiency. Dashed arrow denotes inhibition; solid arrows denote activation

According to PSI theory, the fully functioning person is able to accomplish two main tasks. The first is to achieve volitional efficiency, which means that the person can form explicit plans through reasoned deliberation and put these plans into action (Kuhl & Kazén, 1999). According to PSI theory, volitional efficiency requires that the person can flexibly switch between two personality systems, namely, intention memory and intuitive behavior control. This switching process is schematically displayed in Figure 1. Intuitive behavior control is an elementary personality system that allows the person to execute innate and learned behavioral routines. It is energized by positive affect, which signals that conditions are favorable and the person's needs are being met. When difficulties arise, however, positive affect drops and intention memory becomes activated. Intention memory is a high‐level personality system that inhibits intuitive behavior control, so that the person can stop, figure out what happened, and develop an appropriate action plan. The action plan can be efficiently enacted once positive affect is restored, which reenergizes intuitive behavior control.

**Figure 2:**
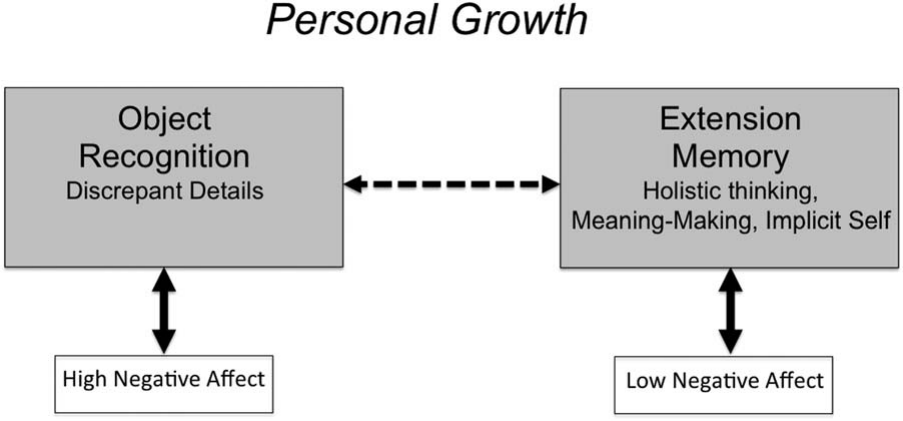
Personality systems interactions for personal growth. Dashed arrow denotes inhibition; solid arrows denote activation

The second main task for the fully functioning person is to achieve personal growth, which means that the person opens up to new (i.e., unexpected or undesired) information and integrates this information in existing networks of autobiographical knowledge (Koole & Kuhl, 2003). According to PSI theory, personal growth requires flexible switching between two personality systems: object recognition and extension memory. This switching process is schematically displayed in Figure 2. Object recognition is an elementary personality system that detects discrepancies between the situation and the person's wishes or expectancies. Object recognition is energized by negative affect, which signals that the situation is potentially threatening. Once the threat has been defused, however, negative affect drops and extension memory becomes activated. Extension memory is a high‐level personality system that generates complex feelings that are based on the simultaneous consideration of the ongoing context and the person's prior memories, values, needs, bodily experiences, and motives. The operation of extension memory is based on high‐level parallel‐distributed processing, which is only partly consciously accessible. Whenever extension memory makes contact with new (often painful) experiences, these experiences can be integrated, so that the knowledge base of extension memory becomes enriched.

Both personal growth and volitional efficiency involve dynamic changes between positive and negative affective states. It thus follows that the ability to flexibly regulate affective states is vital for unlocking the person's full potential (Kuhl & Koole, 2008).

PSI theory proposes two main routes for affect regulation. The first route runs via external support, typically from the social environment. This route is developmentally primary, given that young children need to be soothed and encouraged by their caregiver (Feldman, 2007). Nevertheless, even in adult relationships, social support remains of great value to people when dealing with life demands (Butler & Randall, 2013; Rimé, 2009).

The second route consists of the development of skills for self‐regulating one's own affective states. According to PSI theory, interaction experiences that are sensitive to the person's needs lead to the formation of associative links between extension memory and affect systems, which form the basis for flexible and efficient affect regulation (Kuhl, 2000a). Because not everyone has the good fortune of living in a sensitive environment, individual differences in the ability to self‐regulate affect will emerge. People with well‐developed affect regulation abilities will be better able to act upon their full potential, even in the face of difficulties or stress. PSI hence refers to them as “action‐oriented” people. By contrast, people with less well‐developed affect regulation abilities are likely to become trapped in motivational‐emotional states that keep them from acting upon their personal needs and values. PSI hence refers to the latter as “state‐oriented” people.

#### 2.2.3 Methodology

Given that PSI theory adopts a third‐person perspective on motivation and personality, PSI theorists have concentrated most of their efforts on the development of objective measures of personality functioning. A first class of such objective measures relates to the personality systems that underlie volitional efficiency. PSI theorists have developed several measures designed to tap into intention memory (e.g., Goschke & Kuhl, 1993; Kazén, Kaschel, & Kuhl, 2008). Furthermore, PSI theorists have developed measures to assess the pathway from intention memory to intuitive behavior control, drawing from paradigms in cognitive science (e.g., Petersen & Posner, 2012), such as working memory tasks (e.g., Jostmann & Koole, 2006), the Stroop color‐naming task (e.g., Jostmann & Koole, 2007; Kuhl & Kazén, 1999), and other measures of top‐down action control (Wolff et al., 2016).

A second class of objective measures relates to the personality systems that are relevant to personal growth. PSI theorists have examined several measures designed to tap into the processing characteristics of extension memory, including intuitive judgments of semantic coherence (Baumann & Kuhl, 2002), response latencies for self‐relevant memory retrieval (Kazén, Baumann, & Kuhl, 2004), and congruence between the person's implicit and explicit goals (Baumann, Kaschel, & Kuhl, 2005). In the assessment of implicit (not fully consciously articulated) motives, researchers have developed new kinds of projective tests that are explicitly based on principles from PSI theory (Baumann et al., 2010). Empirical operationalizations of object recognition have received somewhat less attention from PSI theorists. Nevertheless, preliminary findings suggest that error detection (Kazén, Kuhl, & Quirin, 2015) and intrusive thoughts (Baumann & Kuhl, 2003) may be valid indicators of this personality system. Moreover, it may be possible to adapt certain visual perception tasks to measure object recognition (Scheffer & Manke, 2018).

A third class of objective measures relates to affect regulation abilities. According to PSI theory, the most flexible forms of affect regulation operate intuitively, without conscious intention (Koole, Kuhl, Shah, & Gardner, 2008). Consequently, PSI theorists have developed a number of methods to assess intuitive affect regulation. For instance, one task assesses people's latencies of detecting happy faces that are embedded within crowds of angry faces (Koole & Jostmann, 2004, Study 3). Other tasks have used standardized measures of implicit affective processing, such as the affective Simon task (Koole & Jostmann, 2004, Study 2) or affective priming (Koole & Coenen, 2007; Koole & Fockenberg, 2011). Furthermore, PSI theorists have developed an implicit mood measure based on intuitive affective ratings of nonsense words (Quirin, Kazén, & Kuhl, 2009). The latter type of measure has shown good reliability, metric invariance, and construct validity across 10 countries and nine languages, including Chinese, English, Russian, and Spanish (Quirin et al., 2018).

Though their main emphasis has been on objective methods, PSI theorists have also developed self‐report questionnaires. Some of these require people to introspect on their subjective experiences (e.g., mood, personal goals), akin to the questionnaires developed in the SDT tradition. Other questionnaires minimize reliance on introspection by asking people to report on objectively observable behavior. The most widely used questionnaire of the latter variety is the Action Control Scale (ACS), which assesses individual differences in action versus state orientation (Kuhl, 1994; see also Diefendorff, Hall, Lord, & Strean, 2000). Each of the ACS items describes a stressful situation, to which people can respond in either a more action‐oriented manner or in a more state‐oriented manner. Responses are summed across situations to form a score of action versus state orientation. The ACS has two main subscales: The first subscale taps into demand‐related action orientation (also known as decision‐related action orientation) and relates to volitional efficiency. The second subscale taps into threat‐related action orientation (also known as failure‐related action orientation) and relates to personal growth.

#### 2.2.4 Main findings

Aspects of PSI theory have been empirically tested in several hundreds of studies (for a comprehensive review, see Baumann et al., 2018). Though this empirical base is considerable, it remains an order of magnitude smaller than that of SDT. Individual differences in action versus state orientation have been the main focus of PSI research. In line with PSI theory, people high (rather than low) on demand‐related action orientation tend to be more effective in enacting difficult intentions, both in the laboratory (e.g., Jostmann & Koole, 2007; Kazén et al., 2008) and in everyday settings such as education, work, and sports (for a review, see Koole, Jostmann, & Baumann, 2012). Moreover, people high (rather than low) on threat‐related action orientation tend to be more effective in dealing with self‐threatening conditions, such as repeated failure (Brunstein & Olbrich, 1985; Kuhl, 1981), reminders of mortality (Koole & Van den Berg, 2005; Quirin, Bode, Luckey, Pyszczynski, & Kuhl, 2014), unfair situations (IJzerman & Van Prooijen, 2008;Wojdylo, Kazén, Kuhl, & Mitina, 2014), and motivational conflict (Baumann et al., 2005; Hermann & Brandstätter, 2013).

The advantage of action‐oriented people over their state‐oriented counterparts tends to be most pronounced under stressful conditions. This stress‐dependent pattern fits with PSI theory's presumed link between action–state orientation and affect regulation abilities. More direct tests of this idea have shown that action‐oriented people are more efficient in self‐regulating their affective states (as assessed by implicit tasks) than state‐oriented people (Jostmann, Koole, Van Der Wulp, & Fockenberg, 2005; Koole & Fockenberg, 2011; Koole & Jostmann, 2004). Furthermore, the affect‐regulatory effects of action orientation are statistically mediated by increases in extension memory activation (Baumann et al., 2005; Koole & Jostmann, 2004). The latter supports the idea that the self‐regulatory advantages of action‐oriented people derive from improved access to extension memory under stressful conditions.

## 3 INTERFACING SDT AND PSI THEORY

Now that we have characterized SDT and PSI theory separately (see Table 1), we are ready to compare the two theories. In this section, we first discuss the main points at which SDT and PSI theory converge and then turn to where the theories diverge. For each point, we identify some of the underlying theoretical issues and consider how they bear on the question of what it takes to become a fully functioning person.

### 3.1 Convergences

SDT and PSI theory not only cover much of the same conceptual grounds, but the theories also share important assumptions, insights, and empirical interests. It is therefore not surprising that there has been a long‐standing constructive dialogue between SDT researchers and PSI theorists. For instance, in a recent trade book on PSI theory, Julius Kuhl recalled a visit of Richard Ryan to Osnabrück in the 1990s, when they discussed the nature of the self (Storch & Kuhl, 2013, p. 45). Among other things, these discussions resulted in a joint theoretical article (Ryan, Kuhl, & Deci, 1997). Likewise, in a recent Festschrift for Julius Kuhl, Ken Sheldon (2018) shared excerpts of an email exchange between him and Julius Kuhl, which began in 1996 when Sheldon started his SDT‐based research on personal goals and continues to the present day.

Despite these personal exchanges, the common ground between SDT and PSI theory is not readily apparent for people who are new to one or both theories. This is partly due to differences in terminology. Moreover, much of the substantive agreement between SDT and PSI theory lies in shared background assumptions that are only partly articulated. It thus seems useful to make more explicit what the theories have in common. Specifically, we consider three major points of convergence between SDT and PSI theory, which include their (a) emphasis on human agency, (b) adoption of an organismic perspective (see also Ryan, 2018), and (c) positing of dual modes of human self‐regulation.

#### 3.1.1 Human agency

Arguably the most important convergence between SDT and PSI theory is their emphasis on people's capacity to live as free and self‐directed agents. Within SDT, this is reflected in the importance that is attributed to the psychological need for autonomy and the benefits of autonomous self‐regulation. Within PSI theory, this is reflected in the notion that volition represents the highest and most integrative form of personality functioning.

The shared emphasis on freedom and agency by SDT and PSI theory is distinctive, given that other major personality theories have tended to ignore or outright deny human agency. For instance, classical psychoanalysis (Freud, 1922; Westen, 1998) and behaviorist theories proposed that human behavior is driven by elementary drives and habits (Hull, 1943). Likewise, modern social‐cognitive theories of personality have emphasized automatic processes (Bargh & Chartrand, 1999; Greenwald & Banaji, 1995) while downplaying free will (Wegner, 2003). Part of psychologists' resistance against the notion of free will stems from the philosophical view that equates free will with freedom from the laws of causality. However, free will is understood in very different terms within modern scientific accounts, which treat free will as a complex form of action control that is guided by values that are internalized by the self (Baumeister, 2008; Kuhl & Koole, 2004; Ryan & Deci, 2004).

Taken together, both SDT and PSI theory assume that a person can only develop his or her full potential as an autonomous agent who can freely engage with the world. Explicating this freedom poses a formidable scientific challenge. At the same time, this point is of profound applied significance because it means that all psychological interventions should ultimately be aimed at helping people to help themselves. Interventions that try to solve people's problems for them, no matter how well intentioned, are likely to backfire by fostering external dependencies and constraining people's agency.

#### 3.1.2 Organismic paradigm

The second convergence between SDT and PSI theory lies in their joint adoption of the organismic paradigm (Ryan et al., 1997). Science has traditionally sought to explain complex phenomena in terms of simpler material causal principles. However, when the parts of a complex system interact, they may give rise to a new overarching organization (Haken, 2013; Tschacher & Dauwalder, 2003; Vallacher & Nowak, 1994). Recognition of this important principle has led to the organismic paradigm, which originates in biology (Jonas, 1966; Mayr, 1982; see also Ryan et al., 1997). The organismic paradigm can be summarized in three major tenets. First, the organismic paradigm assumes that living beings are active self‐regulating units that are working to maintain and elaborate themselves. Second, the organismic paradigm posits that the organism is composed of higher and lower functional units, which mutually influence another. Third, the organismic paradigm assumes that living organisms have a purpose that originates in their innate needs.

The tenets of the organismic paradigm are clearly discernible in SDT and PSI theory. First, as already discussed, both SDT and PSI theory explicitly recognize the growth‐oriented nature of motivation and personality. Second, the interplay between higher and lower levels is jointly acknowledged by SDT and PSI theory, as signified by the theories' emphasis on self‐determination and volition. Ryan (2018) referred to this as the theories' shared emphasis on the self‐as‐process. Unlike the social‐cognitive tradition, where the self represents an object of one's own perceptions and evaluations (see Morf & Koole, 2012), both SDT and PSI theory have sought to explain the self as the center of experience and as the initiator and regulator of volitional (“self‐determined”) behavior. Third, psychological needs play a key role in both SDT and PSI theory. To be sure, the theories have addressed different aspects of needs: SDT has highlighted how the satisfaction of basic psychological needs forms the bedrock of human motivation (Deci & Ryan, 2000; Prentice, Halusic, & Sheldon, 2014), whereas PSI theory has elaborated how needs may form coalitions with cognitive systems to give rise to implicit motives (Baumann, Kazén, & Kuhl, 2010; Chasiotis & Hofer, 2018; Scheffer & Manke, 2018). Nevertheless, the theories agree on the importance of needs in human motivation and personality.

In sum, SDT and PSI theory agree that the fully functioning person should be understood as a living organism that is a self‐organizing, complex system. Both theories are thus highly compatible with biological approaches to self‐regulation (Di Domenico & Ryan, 2017; Düsing, Tops, Radtke, Kuhl, & Quirin, 2016; see also Gendolla, Tops, & Koole, 2015). At the same time, SDT and PSI theory reject any reduction of the person to lower‐level units, be they genes, drives, or neurons, because such reductions negate the integrative competencies of the person. The latter integrative competencies, according to both theories, are vital for allowing people to reach their full potential.

#### 3.1.3 Dual modes of self‐regulation

**Figure 3:**
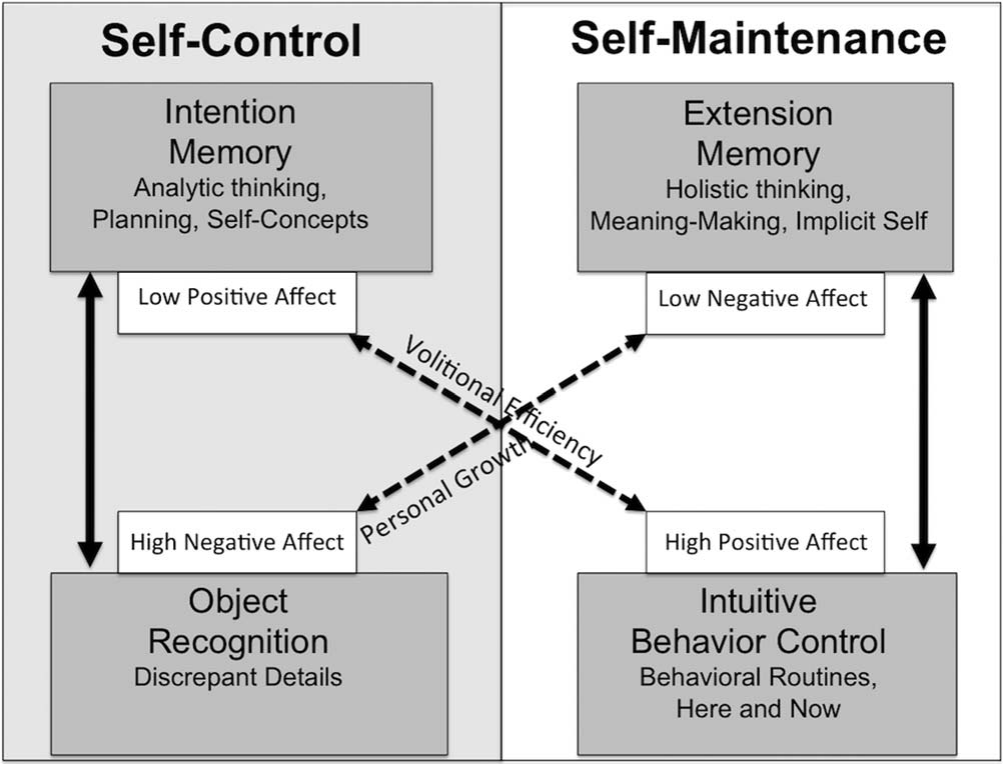
Overview of personality systems interactions (PSI) theory (adapted from Kuhl, 2000a). Dashed arrows indicate inhibition; solid arrows indicate facilitation

The third convergence between SDT and PSI theory lies in their shared recognition of the duality of human self‐regulation. As discussed earlier, SDT distinguishes between autonomous and controlled self‐regulation. This distinction has not yet been reflected in our discussion of PSI theory so far because we only considered the affective modulation of personality systems interactions. In the absence of affective change, however, PSI theory proposes that personality systems form coalitions on the basis of their functional compatibilities (Kuhl, 2000a). These coalitions give rise to two modes of self‐regulation that are similar to SDT's dual modes, as shown in Figure 3.

According to PSI theory, intention memory easily works together with object recognition because both systems operate sequentially, in a step‐by‐step manner. The resulting coalition is referred to as “self‐control.” During self‐control, intention memory ensures that the person engages in problem solving and planning, whereas object recognition ensures that these plans are dutifully executed with precision. Because self‐control is guided by explicit language (see Tullett & Inzlicht, 2010), it is highly receptive to verbal instructions. A person in the self‐control mode will thus be more prone to conform to social expectations and norms about appropriate behavior, analogous to SDT's notion of introjection (Ryan & Connell, 1989). A person in the self‐control mode will further be less inclined to enjoy his or her activities, given that intention memory inhibits positive affect. By contrast, the person will be sensitized to threat of punishment, given that object recognition activates negative affect. The functional profile of self‐control thus corresponds in important respects with SDT's notion of controlled self‐regulation.

According to PSI theory, extension memory easily works together with intuitive behavior control because both systems rely on parallel processing that gives rise to “hunches” or feelings. The resulting coalition is referred to as “self‐maintenance.” During self‐maintenance, extension memory generates complex intuitions and feelings, which guide the person toward self‐congruent actions. The implementation of these actions is supported by the efficient routines of intuitive behavior control, whose execution is energized by positive affect. The latter should not be confused with “mindless” habitual behavior: Self‐maintenance is not guided from the bottom up, by passive stimulus–response chains. Rather, self‐maintenance is directed from the top down, by the person's internalized values, personal narratives, and autobiographically based motives that are part of extension memory. Because extension memory has a broad overview of the person's life experiences, the system can generate remote solutions and action alternatives, leading to flexible and creative behavior. The functional profile of self‐maintenance thus corresponds in important respects with SDT's notion of autonomous self‐regulation.

SDT and PSI theory have thus converged on a similar conception of dual modes of self‐regulation. The implication is that people are often their own worst enemy, given that people's very efforts to control themselves may alienate them from their authentic needs and values, and thus undermine their psychological well‐being. It seems impossible to avoid controlled regulation altogether, given that external pressures are an inescapable part of everyday life. Striking the right balance between the dual modes of self‐regulation thus constitutes an important aspect of becoming a fully functioning person.

### 3.2 Divergences

SDT and PSI theory also diverge in meaningful ways. When theories diverge, it is tempting to assume that where one theory is right, the other must be wrong. However, there are alternative, more integrative ways to resolve theoretical differences. One is to acknowledge that theoretical differences are often simply a matter of perspective. For instance, a classical problem in physics is the question of whether light consists of particles or waves (Kumar, 2011). This matter was eventually resolved within quantum physics, which showed that light has a dual nature that sometimes appears like a particle, and other times like a wave (Heisenberg, 1930). This historical example illustrates how two seemingly contradictory theories may both offer valid insights into different aspects of a complex phenomenon. Given that motivation and personality are highly complex, many theoretical differences in these areas may ultimately come down to a difference in perspective.

Another approach to resolving theoretical differences can be derived from the philosophy of science (Cacioppo, Semin, & Berntson, 2004). Traditionally, scientific theories are seen as approximations of universal truths about reality. Scientific realism has important advantages, by promoting theoretical rigor, verifiability, parsimony, and debate. However, scientific realism is usually not enough to provide satisfactory answers to real‐world problems. Cacioppo and colleagues (2004) have hence proposed that scientific realism may be combined with instrumentalism, or the notion that scientific theories help to answer questions and solve problems in a given domain. Scientific instrumentalism fosters theoretical innovation, synthesis, generativeness, and scope, benefits that are needed to overcome the limitations of scientific realism. An instrumentalist approach consists of asking which different kinds of problems might be resolved by different theories. Instrumentalism may thus also help to reconcile seemingly contradictory theoretical insights.

With these considerations in mind, we turn to three important divergences between SDT and PSI theory. Specifically, SDT and PSI theory diverge in whether they: (a) emphasize a first‐ or third‐person perspective, (b) distinguish between motivation and volition, and (c) view need frustration as potential catalyst of personal growth. In the following paragraphs, we discuss these divergences in more detail. Moreover, to move our discussion in an integrative direction, we consider for each divergence (a) how it may be a matter of perspective, and (b) whether the theories' different insights might speak to different kinds of problems.

#### 3.2.1 First‐ versus third‐person perspectives

The first and arguably most important divergence between SDT and PSI theory lies in the predominant perspectives that they adopt on motivation and personality. SDT emphasizes a first‐person perspective that foregrounds subjective experience. By contrast, PSI theory emphasizes a third‐person perspective that highlights objective competencies.

The distinction between first‐ and third‐person perspectives relates to the question of how objective mechanisms can, in some organisms, give rise to subjective experience. This is also known as “the hard problem of consciousness” (Chalmers, 1997), a theoretical conundrum that, as its name implies, is notoriously difficult to solve. Considered in these terms, it seems all but hopeless to try to bridge the difference between SDT and PSI theory's perspectives. However, it is important to note that the difference in perspectives is not absolute, but relative. Although SDT's primary interest has been in elaborating a first‐person perspective, SDT researchers have also developed and studied objective measures of motivation and personality (e.g., Sheldon et al., 2003; Weinstein, Przybylski, & Ryan, 2013). Conversely, PSI theory's primary interest has been in elaborating a third‐person perspective, but PSI theorists have also developed and studied self‐report questionnaires of motivation and personality (e.g., Baumann et al., 2005; Schlinkert & Koole, in press).

Viewed through an instrumentalist lens, first‐ and third‐person perspectives seem to address different sets of practical problems. A first‐person perspective allows one to empathize with another person, to the point that one can have emotions and sensory experiences similar to those experienced by the person (Batson, Early, & Salvarani, 1997; Macrae, Christian, & Miles, 2014). Such empathy is of tremendous value because it allows researchers and practitioners who are working with SDT to draw upon their intuitions in deriving a scientific analysis of motivation and personality. Although first‐person measures (i.e., self‐report) are inherently subjective, they usually correspond at least to some degree with more objective measures (Sheldon, 2014). A first‐person perspective thus generally remains grounded in objective reality, even though the precise degree of this grounding is bound to remain unclear.

Still, people at times may behave as “strangers to themselves” (Wilson, 2002), who are alienated from their deeper feelings and motivations. This alienation may arise particularly in areas of inner strife and personal difficulty, given that meta‐cognitive abilities tend to be poorest for underdeveloped competencies (Kruger & Dunning, 1999). When people become alienated from the self, a third‐person perspective may prove useful because the latter perspective may provide information about the person that is grounded in objective observations. The more distanced perspective that this affords may generate new insights, especially when it is contrasted with the person's subjective, first‐person perspective. Indeed, researchers and practitioners have developed various procedures for discussing and reconciling the differences between objective and subjective personality measures with clients (Job & Brandstätter, 2009; Storch & Kuhl, 2013; Weber, 2017; see also Kuhl, Kazén, & Koole, 2006).

#### 3.2.2 Distinction between motivation and volition

The second divergence between SDT and PSI theory relates to the distinction between motivation and volition. This distinction is not explicitly recognized by SDT, consistent with the Lewinian tradition (Lewin, 1926; see Gollwitzer, 1993, for a historical review). In the Lewinian tradition, motivated action results directly from the choices that people make on the basis of expectancy–value considerations. People's choices may not be always conscious and deliberate, and people may be deceived into wanting the wrong things for themselves (Sheldon, 2014). Nevertheless, SDT assumes that people, whether they realize it or not, fundamentally have the freedom to choose which path they take and, thereby, which kind of person they become. When people consistently choose activities that promote need satisfaction, their intrinsic motivation and, eventually, their well‐being will rise. When people consistently choose activities to obtain extrinsic rewards like money and fame, or to avoid extrinsic punishments like pain and ridicule, their intrinsic motivation and, eventually, their well‐being will fall. Thus, according to SDT, becoming a fully functioning person is ultimately about making the right choices in life.

PSI theory, by contrast, does distinguish between motivation and volition, in line with Ach's (1905, 1910, 1935) volition psychology. According to PSI theory, people may make all the right choices and feel highly motivated to achieve their personal goals. Nevertheless, when people lack the required volitional competencies, people may still fail to act on their choices. Making the right choices in life is not enough, according to PSI theory. To develop their full potential, people need to master the relevant volitional tasks, such as planning and keeping their spirits up (Kuhl & Fuhrmann, 1998). PSI theory thus holds that becoming a fully functioning person is not just about making the right choices, but also about developing the volitional competencies that are required to implement these choices.

Logically speaking, PSI theory's emphasis on volition is by no means incompatible with SDT. Moreover, from an instrumentalist perspective, there are grounds for believing that volitional processes can be a useful addition to the motivational processes proposed by SDT. For instance, studies have shown that people are more likely to act upon their internalized (or “self‐concordant”) goals—a motivational construct—when these goals are supported by specific action plans—a volitional construct (Koestner, Lekes, Powers, & Chicoine, 2002). Likewise, making specific action plans facilitates goal progress more when people are encouraged to make these plans in an autonomy‐supportive manner (Koestner et al., 2006). Findings such as these suggest that combining the motivational processes proposed by SDT with the volitional processes proposed by PSI theory may allow people to reach their potential more fully than considering motivational or volitional processes separately.

#### 3.2.3 Benefits of need frustration

A third divergence between SDT and PSI theory lies in the significance that they assign to the frustration of psychological needs. Before we delve more deeply into this matter, it should be noted that the two theories agree on many, if not most, important aspects of need frustration: First, SDT and PSI theory agree that need frustration can have adverse effects on motivation and well‐being, especially when people experience frequent and prolonged episodes when their needs are thwarted. Second, SDT and PSI theory agree that people can and often do bounce back from difficult life experiences. Third, SDT and PSI theory agree that such “bouncing back” may increase long‐term resilience by allowing people to acquire new coping strategies and affect regulation competencies. Overall, then, there is considerable agreement on SDT and PSI theory on the psychology of need frustration.

SDT and PSI theory differ, however, in whether they view need frustration as a potential opportunity for psychological growth. According to SDT, need frustration does not provide people with any new possibilities for growth that they could not have had otherwise, in the absence of need frustration. As already noted, SDT does recognize that need frustration may foster resilience, which can be an important boon. However, resilience has a purely defensive function, by allowing people to get back on their feet after having been knocked down by adversity. Resilience is hence not a primary nutrient of growth and wellness. The only way in which people may achieve genuine personal growth—according to SDT—is by satisfying their psychological needs for autonomy, competence, and relatedness, which is the opposite of need frustration.

By contrast, PSI theory posits that need frustration, along with other sources of negative affect, offers a unique—but risky—pathway toward personal growth, provided that people possess sufficient coping resources. According to PSI theory, the harmful effects of need frustration arise only to the extent that people are overwhelmed by the negative affect that accompanies it. When people can overcome this negative affect, these harmful effects should not arise. In fact, in the latter case, need frustration may even be beneficial, by allowing people to develop previously untapped potentials. In this view, people are fundamentally “anti‐fragile” (Taleb, 2012): People do not just have the capacity to resist shocks to their system, but people may actually become better as a direct result of being exposed to manageable stress. According to PSI theory, this is because overcoming need frustration leads the person to switch dynamically between opposing personality systems. This switching process opens a time window for more information exchange between these systems. As a result of such extended information exchange, the person may acquire new kinds of competencies and deepen insights into the self and world.

SDT and PSI theory thus diverge on the question of whether need frustration is a potential catalyst for personal growth. At first glance, the theories seem to directly contradict each other here. Upon closer examination, however, it appears that the theories have a different understanding of what they mean by “personal growth.” Within SDT, personal growth refers to the person's natural tendencies for autonomous self‐regulation (Deci & Ryan, 2000). This conception implies that the person develops tendencies that may lie dormant, but are still inherent in the person's self. In the classic terminology of Piaget (1971; see also Wadsworth, 1996), this process may be classified as “assimilation” because the person fits new information into preexisting schemas and structures within the self. Within PSI theory, this process corresponds with the self‐maintenance mode (see Figure 3).

Personal growth has a different meaning within PSI theory. Specifically, PSI theory regards personal growth as the process whereby higher‐order cognitive structures (e.g., expectancies, meanings) become revised by new information that is not conceivable in terms of the person's existing base of autobiographical knowledge and experiences. In the classic terminology of Piaget (1971), this process of personal development would be classified as “accommodation” because the person is forced to fundamentally alter existing schemas and structures within the self to make room for new information. The type of personal growth described by PSI theory—accommodation—necessitates a confrontation with painful experiences, which makes it qualitatively different from the assimilative type of personal growth described by SDT, which tends to be associated with positive affect.

The classical Piagetian distinction between assimilation and accommodation thus makes it clear that SDT and PSI theory each address a different kind of process when they are referring to “personal growth.” Notably, Piaget (1971) proposed a third conception of personal growth that unites both SDT and PSI theory's conceptions.^1^ Specifically, Piaget suggested that personal growth is fostered by an optimal balance between assimilation and accommodation. The Piagetian perspective thus suggests that SDT and PSI theory's differing conceptions of personality growth may be integrated in an overarching, more encompassing framework of human development.

## 4 THE EMPIRICAL DOMAINS OF INTERNALIZATION, VITALITY, AND FLOW

So far, we have examined SDT and PSI theory on an abstract theoretical level. A deep understanding of the theories, however, can only be obtained by considering the insights that each theory affords into concrete empirical phenomena. In this section, we therefore review how SDT and PSI theory can be brought to bear on recent research in three empirical domains, namely, internalization, vitality, and achievement flow. We chose these domains because they each represent a significant aspect of becoming a fully functioning person. Moreover, the domains are central to the interests of SDT researchers and PSI theorists and, consequently, have been the focus of multiple studies that were guided by each theoretical framework.

Historically, SDT researchers were earlier than PSI theorists to begin their scientific inquiries of internalization, vitality, and achievement flow. We therefore begin our tour of each domain by reviewing the major findings and conclusions of SDT researchers. This is followed by a discussion of work conducted from the perspective of PSI theory. With this narrative sequence, we do not wish to imply that PSI theory should have the last word in each domain. Rather, our narrative reflects that SDT is the more established framework in the respective domains of our review, which places more of a burden on PSI theorists to prove that they have something to add.

### 4.1 Internalization

In everyday life, people frequently have to engage in activities that are not intrinsically motivating, but nonetheless useful, such as house cleaning or filling out tax forms. Through internalization, people develop a willingness to engage in such activities. As discussed before, SDT has distinguished four levels of internalization, ranging from external regulation to introjection, identification, and, finally, integration (see Section 2). According to SDT, people flourish more to the extent that their motivations are more completely internalized. Consistent with this, numerous SDT studies have shown that integrated regulation is accompanied with more intrinsic motivation and greater well‐being, as compared to introjected regulation (for a review, see Sheldon, 2014).

In PSI theory, internalization is the hallmark of personal growth and arises from interactions between object recognition (i.e., recognizing that a task is aversive) and extension memory (the person's extended values and motives). Because these personality systems interactions operate mostly outside of awareness, people may not always realize it when an activity is not fully integrated. When access to extension memory becomes blocked for an extended amount of time, the person can no longer verify whether an activity is sufficiently congruent with the self (Kazén et al., 2003). The person may thus erroneously conclude that the activity is self‐endorsed, even when it is in fact externally imposed, a phenomenon that PSI theory terms “self‐infiltration” (Kuhl & Kazén, 1994). When the person's self becomes infiltrated by external directives, the person is in a state of “latent alienation” (Kuhl & Beckmann, 1994), being unaware of the depth of his or her own alienation.

Kuhl and Kazén (1994) developed an ingenious method to assess self‐infiltration. In this paradigm, participants have to perform a set of tasks, some of which are self‐selected, others of which are assigned by the experimenter. After a filler task, participants receive an unexpected memory retrieval test, in which they are asked to indicate the initial source of each task. The rate of tasks that participants erroneously recall as being self‐chosen, even though the tasks were originally assigned, is taken as an index of self‐infiltration. According to PSI theory, self‐infiltration should increase when people experience persistent negative affect because the latter blocks access to extension memory. Consistent with this, negative mood has been shown to increase self‐infiltration, especially among state‐oriented people, who are less capable of downregulating negative affect (Baumann & Kuhl, 2004). Moreover, state‐oriented people are especially prone to self‐infiltration when they are led to perform aversive activities (Kazén et al., 2003). Finally, self‐infiltrations are associated with chronically elevated levels of the stress hormone cortisol (Quirin, Koole, Baumann, Kazén, & Kuhl, 2009). The latter finding could mean that self‐infiltrations act as a hidden stressor, perhaps by creating inner conflict (see also Baumann et al., 2005).

Taken together, these findings highlight how SDT and PSI theory may complement one another in addressing the dynamics of internalization. SDT offers a comprehensive theoretical analysis of the subjective experiences associated with different levels of integration and their implications for well‐being. Building on and extending this work, PSI theorists have uncovered self‐infiltration as an additional, more covert, form of introjection. Self‐infiltration is unconscious, but it can be reliably detected with an objective measure that capitalizes on memory confusions between self‐selected and assigned activities. SDT and PSI theory thus illuminate different, but complementary, aspects of internalization.

### 4.2 Vitality

Vitality refers to feelings of enthusiasm and of being alive. Energetic constructs like vitality were long neglected, but they have recently made a comeback in personality science (Baumeister, Gailliot, DeWall, & Oaten, 2006; Boksem & Tops, 2008; Thayer, 2003). Contributing to this development, SDT researchers have explained how vitality derives from the dynamics of motivational processes (Ryan & Deci, 2008). SDT holds that autonomously enacted activities, by offering more need satisfaction, should foster vitality more than similar activities that are imposed by external forces. This notion has received converging empirical support (see Ryan & Deci, 2008, for a review). For instance, Kasser and Ryan (1999) found that older adults in a nursing care home experienced greater vitality when they reported more autonomous regulation of their daily activities. Furthermore, older adults reported enhanced vitality when they perceived that their nursing care staff supported their autonomy.

Whereas SDT has emphasized the motivational determinants of vitality, PSI theory suggests that vitality may also be regulated by volitional processes. One important individual difference that relates to volition is action versus state orientation (Kuhl, 1985; Kuhl & Beckmann, 1994). Action‐oriented people tend to be more effective at volitional self‐regulation compared to state‐oriented people. Consequently, action‐oriented people may use volitional processes to shield themselves against the devitalizing impact of demanding situations, for instance, by mentally disengaging from those situations. Such volitional shielding will be harder for state‐oriented people, who may hence be more vulnerable to the devitalizing impact of demanding situations.

Consistent with PSI theory, three recent studies among a total of 971 American participants showed that more demanding life conditions were associated with less vitality among state‐oriented people, but not among action‐oriented people (Schlinkert & Koole, in press). However, this pattern could still have a motivational explanation: It could be that action‐oriented people escape from the devitalizing impact of life demands by simply adopting fewer extrinsic life goals (similar to SDT's construct of an autonomous causality orientation; Deci & Ryan, 1985). Contrary to this, however, action‐oriented people adopted similar (even descriptively higher) levels of extrinsic life goals compared with state‐oriented people when they perceived their lives as more demanding. Moreover, an experimental study showed that action‐oriented people—compared with state‐oriented people—were quicker to mentally disengage from the devitalizing impact of a demanding situation (Schlinkert & Koole, in press, Study 2). These findings fit with the idea that action‐oriented people can draw upon a superordinate process of volitional control to insulate themselves from the depleting effects of demanding conditions (see also Dang, Xiao, Shi, & Mao, 2015; Gröpel, Baumeister, & Beckmann, 2014; Jostmann & Koole, 2007; see Koole et al., 2012, for a review).

### 4.3 Flow

Flow is a motivational state in which people are fully immersed in what they are doing (Csikszentmihalyi, 1990, 2000).

The term was originally coined by Csikszentmihalyi in the 1970s to describe the experiences of people who are engaged in intrinsically motivated activities. According to Csikszentmihalyi and associates, people experience flow when they perceive that they are engaging in challenges that are optimally matched to their capacities, when they have clear goals and receive immediate feedback about goal progress (Nakamura & Csikszentmihalyi, 2002).

Flow tends to be accompanied by marked alterations in awareness, such that people in flow tend to be less aware of themselves and forget that time is passing by.

From SDT's perspective, flow may be regarded as an experiential signature of intrinsic motivation. If this is correct, then flow should arise from the same kinds of conditions that SDT research has shown to be predictive of intrinsic motivation. In line with this, SDT researchers have observed that people experience more flow when they are high in intrinsic motivation and when their psychological needs are being met (Kowal & Fortier, 1999, 2000). Of the three basic needs in SDT—autonomy, competence, and relatedness—satisfaction of competence and autonomy needs is most consistently associated with flow (e.g., Schüler, Brandstätter, & Sheldon, 2013; Schüler, Sheldon, Prentice, & Halusic, 2016). To the extent that flow indexes intrinsic motivation, flow should further be associated with the benefits that SDT ascribes to autonomously motivated activities. Consistent with this, flow is positively associated with well‐being (Bryce & Haworth, 2002) and improvements in academic performance (Heine, 1996), even after controlling for initial ability and grade point average.

Given that flow is accompanied by reduced self‐awareness, people may not be able to report on all the changes within themselves that are connected with a state of flow. The third‐person perspective of PSI theory may thus provide additional insights into the mechanisms of flow. According to PSI theory, flow derives from the dynamic changes in positive affect that arise when people seek out difficulties (which should lower positive affect) and subsequently master these difficulties (which should restore positive affect). (Note that this analysis only pertains to achievement contexts, the focus of most flow research.) PSI theory thus suggests that flow is not based on a fixed state of positive affect from need satisfaction, but rather that flow arises from a dynamic state of alternating between high and low positive affect.

One implication of PSI theory is that flow may be more common among people with contrasting personality traits that are likely to stimulate dynamic alternations between high and low positive affect. This prediction was empirically tested by Baumann and Scheffer (2010, 2011). These researchers focused on two types of traits: first, traits that are associated with inhibited positive affect (i.e., avoidant attachment, schizoid personality style, and introversion), and second, traits that are associated with restoring positive affect (i.e., mastery orientation, and having mastery‐approach goals). As predicted by PSI theory, the combination of both kinds of traits predicted more flow motives, as assessed by a projective measure. The latter measure was also shown to be associated with more flow experiences in everyday life. Moreover, the combination of inhibited positive affect and mastery traits predicted important behavioral indicators of flow, such as improved enactment of difficult intentions in a Stroop task (MacLeod, 1991).

Baumann and Scheffer's (2010, 2011) findings are noteworthy given that avoidant attachment style presumably originates in a developmental history in which the person's relatedness needs were neglected (Mikulincer & Shaver, 2003).

Likewise, schizoid personality style and introversion are associated with less social contact and, hence, lower satisfaction of relatedness needs. Baumann and Scheffer's findings may thus be seen as a first hint that instances of need frustration (in this case, of relatedness needs) may sometimes be beneficial for personality development, provided that people have sufficient coping potential (in this case, mastery orientation).

In sum, SDT has shown how flow—like other manifestations of intrinsic motivation—springs from the satisfaction of psychological needs, especially in the domains of competence and autonomy. Satisfaction of relatedness needs has been less reliably associated with flow in SDT research. The latter may be explained by PSI theory, which suggests that frustration of relatedness needs (e.g., avoidant attachment style) may contribute to flow when it is combined with a mastery orientation. According to PSI theory, frustration of relatedness needs lowers positive affect, and mastery orientation restores positive affect, thereby creating the optimal affective dynamics for flow. The psychology of flow is thus illuminated in different but complementary ways by SDT and PSI theory.

## 5 CONCLUSIONS AND OUTLOOK

In the present article, we have highlighted the integrative potential between SDT and PSI theory. In this last section, we reflect on how this integrative potential may be used to benefit the two theories and personality science at large. Specifically, we consider what each theory can learn from the other in terms of theoretical insights, methods, and applications.^2^ Finally, we discuss what SDT and PSI theory together have to say about what it takes to become a fully functioning person.

### 5.1 What PSI theorists can learn from SDT

One major lesson that PSI theorists can learn from SDT is that a first‐person perspective remains psychologists' primary window into motivation and personality. The first‐person perspective is that of the self‐as‐subject, and thereby indispensable for understanding the person on the person's own terms. Furthermore, SDT's extensive program of research has established that self‐reports, when carefully constructed, have substantial validity in assessing motivation and personality. As Sheldon (2014) observed, self‐report measures may even detect experiential traces of aspects of their personality into which people possess little or no self‐insight. Consequently, PSI theorists would do well to improve their use of the insights afforded by people's self‐reports. For instance, future work by PSI theorists might pay closer attention to experiential correlates of PSI theory's personality systems and develop these into validated questionnaires.

On a methodological level, PSI theorists can learn from SDT researchers' innovative research on within‐person dynamics of motivation and personality processes, for instance, using diary methods and ecological momentary assessments (e.g., Brown & Ryan, 2003; Huta & Ryan, 2010; Milyavskaya et al., 2015). To date, PSI theorists have mainly focused on personality processes that unfold between persons. However, there is ample evidence that personality processes also vary within persons, and that such within‐person variations cannot be reduced to between‐person variations (Cervone, 2005). PSI theory explicitly assumes that the dynamics of personality systems interactions occur within the person. Thus, the theory seems at least, in principle, applicable to the analysis of within‐person dynamics. Addressing within‐person dynamics should thus provide fertile territory for PSI theorists (see Kuhl, Mitina, & Koole, 2017, for initial evidence in this direction).

Finally, when it comes to practical applications like motivational counseling, PSI theorists would do well to heed the central message of SDT, which is that need satisfaction forms the bedrock of healthy personality functioning. To be sure, PSI theory's idea that need frustration may set the stage for personal growth could provide great hope to people in need‐thwarting environments. However, even if this idea turns out to be completely correct, it at best represents a very risky pathway to personal growth. According to PSI theory, need frustration should only foster personal growth when it does not exceed the person's coping potential. When the person's coping potential is overtaxed by prolonged need frustration, the person will likely pay a steep price, in terms of significant reductions in well‐being and mental health. Thus, a great deal more research is needed into need frustration as a potential pathway to personal growth before this idea can be responsibly put into practice. For the time being, SDT's emphasis on need satisfaction provides the most solid scientific basis for motivational interventions.

### 5.2 What SDT researchers can learn from PSI theory

Conversely, SDT researchers may learn a thing or two from PSI theory. One eye‐opener for SDT researchers may be the notion that it is possible to develop a third‐person perspective on motivation and personality without reverting to reductionism. The association between a third‐person perspective and reductionism has historically been the main reason why many humanistically oriented researchers have shied away from developing a third‐person perspective on psychology. PSI theory suggests, however, that a third‐person perspective can be reconciled with the tenets of a nonreductionist, organismic paradigm. This may embolden SDT researchers to study SDT processes from a third‐person perspective. Such a development could help to refute recurring claims that SDT constructs like autonomy are illusory (see also Ryan & Deci, 2004), by highlighting how SDT processes are in fact grounded in objective reality and readily observable outside the sphere of the person's lived experience.

Methodologically, a third‐person perspective could open up an entire range of new research tools for SDT researchers. These measures could directly benefit from the kinds of techniques that have so far been developed by PSI theorists, such as intention memory tasks (e.g., Goschke & Kuhl, 1993; Kazén et al., 2008), affect misattribution procedures (e.g., Quirin, Bode, & Kuhl, 2011; Quirin, Kazén, et al., 2009), response time tasks (e.g., Kazén et al., 2003; Koole & Jostmann, 2004), projective tests (Baumann et al., 2010; Scheffer, Eichstaedt, Chasiotis, & Kuhl, 2007), motive priming (Kazén & Kuhl, 2005; Kuhl & Kazén, 2008), and cognitive control tasks (Jostmann & Koole, 2006, 2007; Kuhl & Kazén, 1999). Of particular interest may be the questions of whether and how providing people with feedback on objective personality measures may promote self‐insight and personal growth, as would be expected on the basis of SDT and PSI theory (see Kuhl et al., 2006).

In translating SDT's insights into practical interventions, it may be useful to consider PSI theory's notion that healthy personality functioning is not just a matter of choosing the right goals to pursue, but also one of buttressing those goals with the relevant volitional competencies. As discussed before, there is reason to believe that volitional factors may interact synergistically with the motivational processes proposed by SDT, for instance, by helping people to act upon their autonomously motivated goals (Koestner et al., 2006, 2002) or by shielding people from autonomy‐undermining influences (Koole, 2004; Schlinkert & Koole, in press). Practitioners may thus enhance the effectiveness of SDT‐based interventions by ensuring that they are implemented among populations with adequate volitional competencies. Among populations with inadequate volitional competences, SDT‐based interventions may be complemented by volitional training exercises.

### 5.3 Developing a person's full potential: Insights from SDT and PSI theory

We opened this article with some observations of the beloved humanistic psychologist Carl R. Rogers (1961) on the challenges of becoming a fully functioning person. Now that we have examined and compared SDT and PSI theory, it has become apparent how the two theories address these challenges in mutually compatible ways.

To allow a person to develop his or her full potential, both SDT and PSI theory suggest that it is vital to nurture the person's capacity for autonomous, volitional action control. Such nurturance, according to the two theories, presumes that the person's social environment is supportive and sensitive to the person's psychological needs. According to SDT, supportive social interactions foster need satisfaction, thereby allowing the person's natural capacities for growth to emerge (Deci & Ryan, 2000). According to PSI theory, sensitive social interactions foster the person's capacity for intuitive affect regulation (Koole & Jostmann, 2004), which provides the basis for the person's volitional competencies (Kuhl, 2000a).

Both SDT and PSI theory suggest that the person can only realize his or her full potential when he or she can successfully internalize outside norms and expectancies. According to SDT, internalization is facilitated by the social environment when it acknowledges the person's feelings, and by dispositional factors, such as autonomy orientation, that support autonomously motivated behavior. SDT thus emphasizes choice and motivation. According to PSI theory, internalization is facilitated by affect regulation, either through social support or through self‐regulation. PSI theory thus emphasizes emotional maturity and volitional competencies. In short, SDT and PSI theory offer a wealth of overlapping and complementary recommendations for helping the individual to develop into a fully functioning person. In the spirit of Rogers (1961), we hope that this work will encourage people to live up to their full potential, so that people may become who they are.

## CONFLICT OF INTERESTS

The author(s) declared no potential conflicts of interest with respect to the research, authorship, and/or publication of this article.

## ENDNOTES

1 We thank Julius Kuhl for alerting us to this Piagetian integration.

2 We thank an anonymous reviewer for suggesting this integrative way of concluding the present article to us.
